# Constructing Nanocaged Enzymes for Synergistic Catalysis of CO_2_ Reduction

**DOI:** 10.1002/advs.202300752

**Published:** 2023-05-10

**Authors:** Zhichao Jia, Jianan Dang, Guobin Wen, Yanxing Zhang, Zhongwei Chen, Zhengyu Bai, Lin Yang

**Affiliations:** ^1^ Collaborative Innovation Center of Henan Province for Green Manufacturing of Fine Chemicals Key Laboratory of Green Chemical Media and Reactions Ministry of Education School of Chemistry and Chemical Engineering Henan Normal University Xinxiang Henan 453007 P. R. China; ^2^ Department of Chemical Engineering Waterloo Institute for Nanotechnology University of Waterloo 200 University Avenue West Waterloo ON N2L 3G1 Canada; ^3^ School of of Physics Henan Normal University Xinxiang Henan 453007 P. R. China

**Keywords:** bioelectrocatalytic reaction, CO_2_ reduction, formate dehydrogenase, hydrophobicity, nanocage structure

## Abstract

Promoting the activity of biological enzymes under in vitro environment is a promising technique for bioelectrocatalytic reactions, such as the conversion of carbon dioxide (CO_2_) into valuable chemicals, which is a promising strategy to address the environmental issue of CO_2_ in the atmosphere; however, this technique remains challenging. Herein, a nanocage structure for enzyme confinement is synthesized to enable the in situ encapsulation of formate dehydrogenase (FDH) in a porous metal–organic framework, which acts as a coenzyme and boosts the hybrid synergistic catalysis using enzymes. This study reveals that the synthesized FDH@ZIF‐8 nanocage‐structured hybrid (CSH) catalyst exhibits an improved catalytic ability of the enzymes and increases the hydrophobicity of the electrode and its affinity to CO_2_. Thus, CSH can trap CO_2_ and control its microenvironments. The CSH catalyst boosts the conversion rate of CO_2_ to formic acid (HCOOH) to 28 times higher than that when using pure FDH. The in situ attenuated total reflectance surface‐enhanced infrared absorption spectroscopy (ATR‐SEIRAS) spectra indicates that OCHO* is the key intermediate. Density functional theory (DFT) calculations show that CSH has extremely low overpotential and is particularly effective for producing formate. This protection architecture for enzymes considerably promotes their biological application under in vitro environments.

## Graphical abstract

An in vitro construction of a cage‐structured hybrid (CSH) catalyst is proposed to stabilize the formate dehydrogenase (FDH), and the 2‐methylimidazole groups in CSH play a vital role similar to that of a coenzyme, which is connected to the active site (valine) of the enzyme via the hydrogen bond and promotes the activity of FDH enzymes.

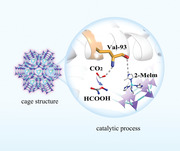

## 1 Introduction

Converting carbon dioxide (CO_2_) into high‐value chemicals using electrochemical CO_2_ reduction reactions (CO_2_RRs) is an attractive and promising process for realizing carbon neutrality. However, the application of this process depends on the further development of active and stable catalytic materials.^[^ ^1^ ^]^ The unique catalytic properties and high efficiency of enzymes provide opportunities for growth in the field of energy catalysis beyond the current metal‐based catalysts.^[^ ^2^ ^]^ Enzymes are the key catalysts in living cells that enable complex transformations in organisms.^[^ ^3^ ^]^ However, the inherent fragility of isolated enzymes outside the membranes of living cells causes operational difficulties and their easy deactivation.^[^ ^4^ ^]^ Therefore, the instability of enzymes in abiotic conditions limits their application in the field of energy catalysis.^[^ ^5^ ^]^ Thus, an efficient artificial cell structure should still be developed to protect the enzyme and obtain biological enzyme materials with a high conversion rate and selectivity.^[^ ^6^ ^]^

Formate dehydrogenase (FDH) is an enzyme that exhibits high efficiency and selectivity in *Candida boidinii* and could convert carbon sources into biofuels.^[^ ^7^ ^]^ Nevertheless, the reaction rate of FDH is affected by environmental conditions such as pH, temperature, and hydrophobicity.^[^ ^8^ ^]^ Some amino acids, such as valine, in the enzymes can affect the hydrophobicity around the active pocket of enzymes,^[^ ^9^ ^]^ enhancing their activity.^[^ ^10^ ^]^ Constructing a bionic cell membrane around the enzymes can be used for their protection under in vitro environment, such as using porous materials as a functional host.^[^ ^11^ ^]^ Metal–organic frameworks (MOFs), such as FDH@NU‐1006^[^ ^7b^ ^]^ and GOx@MOF,^[^ ^12^ ^]^ are crystalline porous materials^[^ ^13^ ^]^ that can store chemical species for further reaction.^[^ ^14^ ^]^ Therefore, a host was designed herein to cage FDH and valine, forming a composite catalytic system for CO_2_ reduction.

Herein, a synergistic architecture was designed between the protective structure and the enzyme to further promote the enzymatic activity. A FDH@ZIF‐8 nanocage‐structured hybrid (CSH) catalyst was constructed through the in situ generation of ZIF‐8 around FDH at room temperature. The CSH catalyst provides an in vitro hydrophobic microenvironment with coenzyme‐like structures for FDH. The conversion rate of CO_2_ to formic acid (HCOOH) using the developed CSH catalyst reached 103.9 mM h^−1^, which is ≈28 times higher than that of pure FDH. in situ attenuated total reflectance surface‐enhanced infrared absorption spectroscopy (ATR‐SEIRAS) spectroscopy indicates that OCHO* is a critical intermediate in the conversion of CO_2_ into HCOOH. Simulation studies show that the 2‐methylimidazole (2‐Melm) groups from ZIF‐8 play a vital role similar to that of a coenzyme, connecting it to the active site of the enzyme (valine), which promotes enzyme's activity. The hydrophobic microenvironment in the MOF cavity can improve the affinity of the enzyme for CO_2_.

## 2 Results and Discussion

**Scheme 1:**
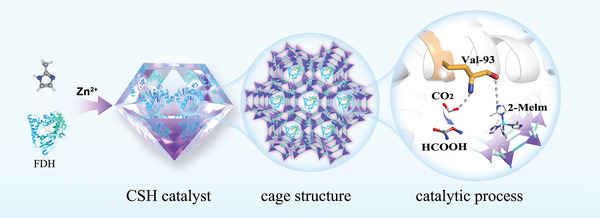
Schematic of the preparation of the CSH catalyst.

**Scheme** **1** shows the formation of the CSH catalyst. First, the 2‐Melm and FDH molecules were combined via intermolecular interactions. The ligand‐binding is promoted by the addition of divalent zinc cations. The CSH catalyst was then formed so that the FDH molecules were located in the cavities of the ZIF‐8 nanocage structures. Valine 93 of FDH, which is the active site, was connected to the 2‐Melm ligand of ZIF‐8. At the same time, FDH, was encapsulated in situ via MOFs to immobilize the enzymes under cell‐free conditions and build artificial cell structures with a biological microenvironment.

**Figure 1:**
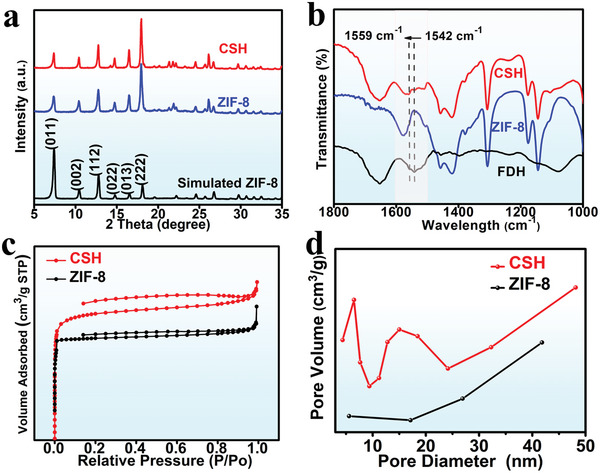
a) XRD patterns of the CSH catalyst, ZIF‐8, and simulated ZIF‐8. b) FT‐IR spectra of the CSH catalyst, ZIF‐8, and FDH. c) N_2_ adsorption isotherms on ZIF‐8 and the CSH catalyst. d) Pore size analysis of ZIF‐8 and the CSH catalyst.

To determine the composition and analyze the crystal structure of the targeted sample, a powder X‐ray diffraction (XRD) test was performed. **Figure** **1**a clearly shows the diffraction peaks of ZIF‐8 and the CSH catalyst, which are consistent with the standard card.^[^ ^15^ ^]^ The diffraction peaks observed at 7.4°, 10.4°, 12.8°, 14.7°, 16.5°, and 17.9° can be attributed to the reflection planes of (011), (002), (112), (022), (013), and (222) of ZIF‐8, respectively. This suggests the successful synthesis of ZIF‐8. The Fourier Transform Infrared (FT‐IR) spectrum was used to further confirm that the 2‐Melm and FDH molecules are combined (Figure 1b). FDH exhibits a prominent infrared characteristic absorption peak at 1542 cm^−1^, which is primarily explained by the protein's N—H bending and C—N stretching vibrations, corresponding to the amide bond stretching vibration.^[^ ^16^ ^]^ Figure 1b shows that the characteristic absorption peak position of CSH blue shifts to 1559 cm^−1^, confirming the interaction between the FDH and the 2‐Melm molecules.

The Nitrogen (N_2_) adsorption isotherm was measured at 77 K. The BET area decreased from 409.6 to 231.6 cm^2^ g^−1^ because FDH occupied part of the pores of the MOFs. The CSH catalyst has mesopores (6.5 and 15.0 nm, Figure 1d). The CSH catalyst is selected as the supporting material because the particle size of FDH^[^ ^7^ ^]^ (6 nm × 4 nm × 11 nm) matches the size of the mesoporous channels (6.5 and 15.0 nm). This further suggests the successful integration of FDH into the ZIF‐8 structure. The pore structure can increase the contact between the CSH catalyst and the electrolyte, which is conducive to the charge transfer.^[^ ^17^ ^]^ To further study the CO_2_ adsorption performance of the CSH catalyst, the CO_2_ absorption isotherms of the CSH catalyst, ZIF‐8, and FDH were obtained at 273 and 298 K (Figure S1, Supporting Information). The CSH could adsorb CO_2_ (22.8 cm^3^ g^−1^) at 273 K and (13.3 cm^3^ g^−1^) at 298 K, which is more than FDH (1.1 cm^3^ g^−1^) at 273 K and (1.3 cm^3^ g^−1^) at 298 K. In addition, ZIF‐8 could adsorb CO_2_ (26.3 cm^3^ g^−1^) at 273 K and (11.2 cm^3^ g^−1^) at 298 K. The results show the CSH catalyst exhibits a better adsorption capacity for CO_2_ than FDH. The varying surface areas and CO_2_ affinities of the three materials can be used to explain this variation in the amount of CO_2_ adsorption. In the thermogravimetric (TG) analysis diagram (Figure S2, Supporting Information), the weight loss of the CSH catalyst and ZIF‐8 are 6.6% and 1.9% at 100 °C, respectively. This can be attributed to the loss of the adsorbed water in the catalysts. At 220–600 °C, the weight loss of the CSH catalyst and ZIF‐8 are 5.3% and 3.2%, respectively. This difference is attributed to the decomposition of the enzyme to achieve the FDH encapsulation. The absorbance of FDH at 562 nm by a microplate reader working within the ultraviolet (UV) range was used to calculate the encapsulation efficiency of the enzyme during the synthesis process, which reached 51.4% (Figure S3, Supporting Information). To prove the hydrophobicity within the pores of CSH, water adsorption tests are added for the CSH and FDH@ZIF‐90. The pores of CSH contain hydrophobic groups (‐CH_3_), and the pores of FDH@ZIF‐90 contain hydrophilic groups (—OH).^[^ ^18^ ^]^ The water adsorption values of CSH and FDH@ZIF‐90 are 1.5 and 7.0 mmol g^−1^, respectively (Figure S4, Supporting Information). The CSH could adsorb N_2_ (165.8 cm^3^ g^−1^), which is more than FDH@ZIF‐90 (29.2 cm^3^ g^−1^) (Figure S5, Supporting Information). In addition, the CSH could adsorb CO_2_ (13.3 cm^3^ g^−1^) at 298 K, which is more than FDH@ZIF‐90 (6.3 cm^3^ g^−1^) at 298 K (Figure S6, Supporting Information). A greater extent of adsorption of nonpolar gases indicates the pores more hydrophobic.^[^ ^19^ ^]^ The results suggest that the CSH catalyst forms a hydrophobic microenvironment.

**Figure 2:**
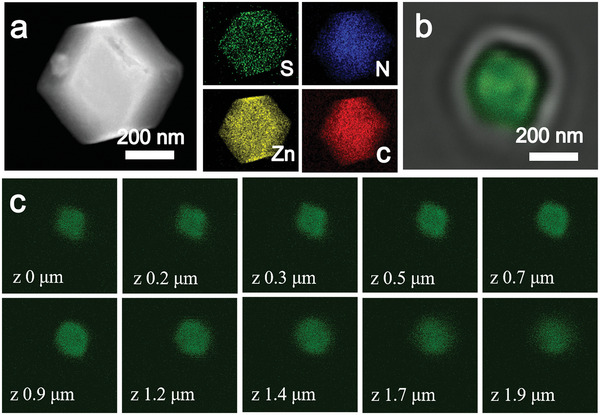
a) HAADF‐STEM of the CSH catalyst and EDS‐mapping images of S, N, Zn, and C. b) CLSM diagram of the CSH catalyst, and c) confocal images of the CSH catalyst with different z‐axis focal planes.

Scanning electron microscope (SEM) and transmission electron microscopy (TEM) were used to characterize the polyhedron structure of the CSH catalyst, exhibiting regular shape and uniform size distribution of the catalyst. The SEM image shows that the CSH catalyst particles had a dodecahedral structure and their average size was 600 nm (Figure S7, Supporting Information), which is consistent with the size of ZIF‐8 (Figure S8, Supporting Information). **Figure** **2**a shows the internal structure of the CSH catalyst. Some defects are observed within the CSH material (Figure S9, Supporting Information). These results show that FDH addition affects the crystallinity and uniformity of ZIF‐8. The energy dispersive X‐ray spectrometry (EDS) mapping images show the uniform distribution of S, N, Zn, and C within the CSH material. Moreover, they confirm that FDH and ZIF‐8 are well combined. To verify that FDH was successfully encapsulated into the cavities of ZIF‐8, the CSH catalyst was studied using the confocal laser scanning fluorescence microscopy (CLSM). The catalyst was dipped in a fluorescein isothiocyanate (FITC) solution, which can precisely coordinate with FDH and emit green fluorescence under light excitation at 405 nm. The CLSM image (Figures. 2b; Figure S10, Supporting Information) indicates that the entire CSH catalyst stained with FITC emits apparent green fluorescence under light excitation at 405 nm.

Figure 2b shows that the CSH shell does not emit any green fluorescence, whereas the interior of the CSH catalyst clearly does, suggesting that the external adsorption of the loose enzyme is washed out. The FITC and CLSM analyses revealed the ZIF‐8 with no FDH (Figure S11, Supporting Information) and FDH stained with FITC (Figure S12, Supporting Information), respectively. The results verify the encapsulation of FDH in the ZIF‐8 channel. Laser confocal slice cutting was performed to further prove the distribution of FDH in ZIF‐8. Figure 2c shows the confocal images of different z‐axis focal planes of the CLSM images of the CSH catalyst. This figure indicates that the green fluorescence follows a shallow–deep–shallow pattern with the change in the confocal plane in the z‐direction. This trend indicates that the FDH molecules are located inside the ZIF‐8 channels.

**Figure 3:**
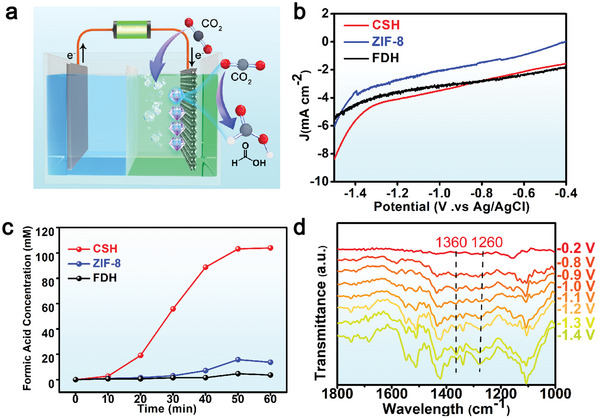
a) Schematic of the bioelectrocatalytic CO_2_ reduction device. b) LSV scans of the CSH catalyst, ZIF‐8, and FDH measured in CO_2_‐saturated PBS buffer (10 mM). Scan rate = 5 mV s^−1^. c) Concentration of HCOOH generated by the CSH catalyst, ZIF‐8, and FDH in phosphate‐buffered saline (PBS) saturated with CO_2_. d) In situ ATR‐SEIRAS spectra of the CSH catalyst from −0.8 to −1.4 V versus Ag/AgCl in CO_2_‐saturated PBS buffer (10 mM).

An electrocatalytic CO_2_ reduction device was built to study the conversion efficiency of CO_2_ to HCOOH when using the CSH catalyst (**Figure** **3**a). The CSH catalyst was deposited on the carbon cloth electrode, and CO_2_ gas was continuously blown into the H‐type electrolytic cell. Figure 3b shows the linear sweep voltammetry (LSV) curves of the CSH catalyst, ZIF‐8, and FDH, which are tested under the conditions of CO_2_ saturation. The current for the CSH catalyst is considerably higher than those for ZIF‐8 and FDH. This result confirms that the CSH catalyst exhibits catalytic properties for CO_2_ conversion. In addition, the concentration gradient of a HCOOH solution was configured, and a HCOOH standard curve was obtained using high‐performance liquid chromatography (Figure S13, Supporting Information). The test was conducted at an applied potential of −1.1 V versus Ag/AgCl for 60 min, and the CO_2_ conversion to HCOOH using the CSH catalyst reached 103.9 mM h^−1^ (Figure 3c), which is considerably higher than that when using pure FDH (3.6 mM h^−1^), ZIF‐8 (13.7 mM h^−1^), and FDH@ZIF‐90 (9.7 mM h^−1^) (Figure S14, Supporting Information), indicating that the hydrophobic microenvironment is beneficial to the catalytic activity of FDH. No HCOOH was generated when a pure carbon cloth electrode was used, indicating that HCOOH was not introduced by the carbon cloth (Figure S15, Supporting Information). Furthermore, the CSH electrode did not produce HCOOH when tested under N_2_ conditions (Figure S16, Supporting Information). Because the enzyme involved an activation process, the reaction rate was slow in the first 10 min, and then, the reaction rate gradually increased. After 30 min, the nicotinamide adenine dinucleotide (NADH) coenzyme was slowly consumed, and the reaction rate decreased (Figure 3), which is consistent with the results of a previous study.^[^ ^20^ ^]^ The catalytic efficiency of the CSH catalyst for reducing CO_2_ to HCOOH was ≈28 times higher than that of pure FDH. This is a typical reversible electrochemical catalysis process:^[^ ^21^ ^]^

(1) $${\mathrm{CO}}_{2}+\mathrm{NADH}+H^{+}⇌\mathrm{HCOOH}+{\mathrm{NAD}}^{+}$$

The catalytic direction of FDH depends on the redox potential of the coenzyme, which can be influenced by the pH and the concentration of substrates and products. In general, at low pH and high concentrations of formate and NAD^+^, the reaction proceeds in the direction of formate oxidation to CO_2_, while at high pH and low concentrations of formate and high concentrations of NADH, the reaction proceeds in the direction of CO_2_ reduction to formate.^[^ ^22^ ^]^

In situ ATR‐SEIRAS spectroscopy was performed at room temperature between 1000 and 1800 cm^−1^ to determine the electrocatalytic reaction path of the catalyst. A previous ATR‐SEIRAS study confirmed that the peak observed at ≈1360 cm^−1^ is the characteristic peak of the stretching vibration of OCO in the two oxygen‐bridged formate species (OCHO*).^[^ ^23^ ^]^ Figure 3d shows the in situ ATR‐SEIRAS spectroscopy results of the CSH catalyst at different potentials. The intensity of the peak observed at ≈1360 cm^−1^ gradually increases with the increasing applied potential. This confirms the formation of OCHO* as an intermediate in the reaction process of the electrochemical CO_2_RR using the CSH catalyst. Gas chromatography/mass spectrometry (GC‐MS) characterization of the key intermediate was performed in Figure S19 (Supporting Information). Based on the resulting total ion flow chromatogram, the ion extraction peaks of CO_2_ and HCOOH are identified (Figure S17a, Supporting Information), with the characteristic ion mass‐to‐charge ratios (m/z) of HCOOH being 44, 45, 46, and 47, and the m/z 45 may be OCHO* (Figure S17b, Supporting Information).^[^ ^24^ ^]^ These findings suggest that the HCOOH ions are present in the sample at these specific m/z values. These results provide valuable information for identifying and quantifying HCOOH in the sample. They could be used to improve the sensitivity and accuracy of analytical methods for detecting HCOOH. Through electron spin resonance (ESR) analysis, the detection of formate radicals can provide proof of intermediates (Figure S18, Supporting Information),^[^ ^25^ ^]^ offering further evidence to support the reaction mechanism.

**Figure 4:**
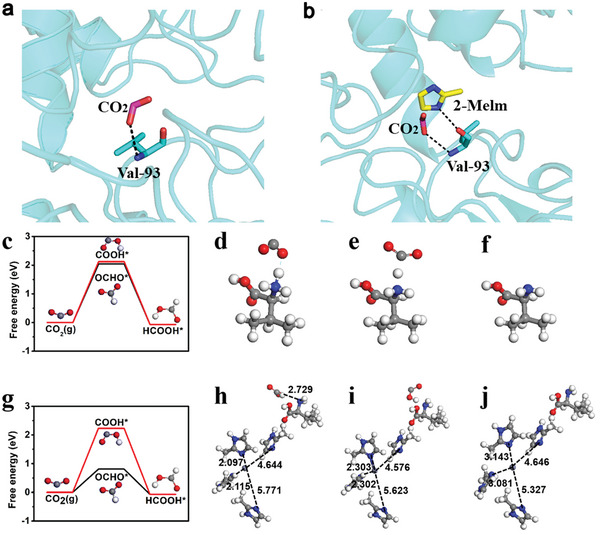
a,b) 3D structural alignment of FDH. c,g) Free‐energy diagrams of HCOOH* generated using FDH and the CSH catalyst. The optimized FDH and CSH catalyst (d,e,h,i) with and (f,j) without COOH* and OCHO* adsorptions. The gray, white, blue, purple, and red balls represent C, H, N, Zn, and O, respectively.

The AutoDock Vina software and theoretical calculations were used to simulate the reaction process to further verify the reaction mechanism of the CSH catalyst. The atomic coordinates of *Candida boidinii* FDH (PDB: 5DN9) were obtained from the Protein Data Bank.^[^ ^10b^ ^]^ The nuclear coordinates of 2‐Melm were downloaded from PubChem, and the energy was minimized using the chem3D software. The AutoDock Vina software was used and the lowest energy complex was chosen to build the complex of *Candida boidinii* FDH and 2‐Melm.^[^ ^26^ ^]^ Pymol (http://www.pymol.org/) was used to prepare structural images of FDH and 2‐Melm. The valine around the deep pockets of FDH was connected to CO_2_ by hydrogen bond (**Figure** **4**a). The valine around the pockets of FDH was related to CO_2_ and 2‐Melm at the same time (Figure 4b). Numerical calculations^[^ ^27^ ^]^, such as molecular docking and molecular dynamics simulations, were used to reveal the possible promotion mechanism of the 2‐Melm group. These calculations provide a theoretical basis for the interaction between FDH and small molecules.

Models of the valine and 2‐Melm composites were constructed to reveal the high efficiency of the CO_2_ conversion to HCOOH (Figure 4f,j). The rate‐limiting overpotential of HCOOH formation was calculated using the standard hydrogen electrode method developed by Nørskov et al.^[^ ^28^ ^]^ The exact computational details are included in the computational methods section. The adsorption energy of CO_2_ on valine and 2‐Melm composite models (0.21 eV) is slightly larger than valine (0.17 eV) (Figure S19, Supporting Information). Figure 4c,g shows the free‐energy diagrams of HCOOH generated using the valine and 2‐Melm composite, respectively. The final state of HCOOH in Figure 4c,g is adsorbed state denoted as HCOOH*, and the calculated Δ G for the formation of HCOOH* is −0.07 eV. Figure 4d,e,h,i show the detailed COOH* and OCHO* adsorption configurations. Compared with the overpotential of the valine for HCOOH (2.02 eV), valine and 2‐Melm composite has a lower overpotential (0.81 eV), especially based on the OCHO* path. The ligands of 2‐Melm can effectively tune the catalytic activity of the valine. After the adsorption of OCHO*, 2‐Melm in the valine–2‐Melm composite shifts to a position considerably closer to the Zn‐ion center. Figure s. 4 h and 4j show that the bond length between two 2‐Melm units and Zn decreased by ≈1 Å (from 3.14 and 3.08 Å to 2.09 and 2.11 Å, respectively). This can help lower the free energy of OCHO* adsorbed on the amic acid–2‐Melm composite and reduce its overpotential for HCOOH production.

## 3 Conclusion

FDH is successfully encapsulated in the developed porous nanocage structure, providing an in vitro favorable microenvironment for enzymatic reactions. The catalyst prepared in situ exhibits excellent catalytic activity. Moreover, the porous absorbent structure exhibits excellent CO_2_ adsorption, contributing to the conversion of CO_2_ into HCOOH. The results confirmed that the hydrophobicity around the protein of biological macromolecules should be controlled and their biological microenvironment should be regulated to promote and retain their natural function. These results show the potential for developing efficient and sustainable carbon‐fixation reaction systems and shed light on the construction of biocatalysts with good biological activity.

## 4 Experimental Section

### Materials and Methods

Formate dehydrogenase from Candida boidinii (CAS number: 9028‐85‐7, catalog number: F8649‐50UN) was produced from Sigma‐Aldrich, USA. NADH (molar mass: 709.4, CAS number: 606‐68‐8 (anhydrous), part number: MFCD00036200), potassium dihydrogen phosphate, sodium chloride, potassium chloride, and disodium hydrogen phosphate were produced from Macklin, China. Zinc acetate and zinc nitrate hexahydrate was made from Sinopharm Chemical ReagentCo., Ltd, China. 2‐Melm was created from Xiya Reagent, China. Sodium nitrate and neutral red were produced from Tianjin Kemiou Chemical Reagent Co., Ltd., China. Imidazole‐2‐formaldehyde (2‐ICA) was produced by Shanghai Maclin Biochemical Technology Co., Ltd., China.

### Synthesis of the CSH Catalyst

CSH was synthesized by mixing zinc acetate, FDH, and 2‐Melm in an aqueous solution. First, 300 mg 2‐Melm was dispersed in 4 mL deionized water. Next, 5 mg FDH was added and stirred for 2 min. 30 mg zinc acetate was dispersed into 1 mL deionized water. The combined solution was then swirled at room temperature for 2 h. The white result, marked as CSH, was then centrifuged three times in deionized water before being dried in room air.

### Synthesis of ZIF‐8

ZIF‐8 was synthesized by mixing Zinc acetate and 2‐Melm in an aqueous solution. The reaction conditions were the same as the CSH catalyst except without FDH.

### Synthesis of FDH@ZIF‐90

As the contrast experiment, FDH@ZIF‐90 was synthesized by mixing 2‐ICA, FDH, and zinc nitrate hexahydrate in an aqueous solution. First, 60 mg 2‐ICA was dispersed into 4 mL deionized water and heated at 40 °C to dissolve it quickly. Next, 5 mg FDH was added into the system under vigorous stirring for 2 min. And then, 1 mL deionized water was combined with 50 mg zinc nitrate hexahydrate for 30 min. Finally, the precipitate was washed with deionized water by centrifugation three times and dried in ambient air.

### Characterization

N_2_ and CO_2_ adsorption isotherms were measured on Micromeritics ASAP 2460. Adsorption isotherms of H_2_O were measured on a Quantachrome AUTOSORB‐1 volumetric gas adsorption analyzer. The XRD measurement was performed on a PANalytical B.V. X, Pert3 Power powder diffractometer. SEM images were collected on Hitachi SU8010. TEM images and EDS were collected on ThermoFisher Scientific FEI Talos F200. FT‐IR spectrum was recorded on a Shimadzu IR‐100 spectrometer with a wavenumber range of 4000–400 cm^−1^. The CLSM was performed on Leica TCS SP8. The TG analysis was performed on a Netzsch STA449C instrument. The contact angle test was performed on KRUSS DSA25. The HCOOH was measured by Agilent HP‐1100 high‐performance liquid chromatography. The samples were derivatized before each measurement. FDH encapsulation was measured by a multimode plate reader (PerkinElmer, USA). Agilent 7890B‐7000D was used for GC‐MS analysis. On a Bruker X‐band A200 spectrometer, the EPR spectra of formate radicals was captured.

## Conflict of Interest

The authors declare no conflict of interest.

## Supporting information

Supporting Information

## Data Availability

The data that support the findings of this study are available from the corresponding author upon reasonable request.
