# Supplementary material for: Constructing Nanocaged Enzymes for Synergistic Catalysis of CO2 Reduction
Source: Adv Sci (Weinh). 2023 May 10;10(20):2300752. doi: 10.1002/advs.202300752 (PMC10369272; doi:10.1002/advs.202300752)
Supplement: Supplementary file 1 — Supporting Information [file ADVS-10-2300752-s001.pdf]

ADVANCED SCIENCE
Open Access

Supporting Information

for *Adv. Sci.*, DOI 10.1002/advs.202300752

Constructing Nanocaged Enzymes for Synergistic Catalysis of CO_2 Reduction

Zhichao Jia, Jianan Dang, Guobin Wen, Yanxing Zhang, Zhongwei Chen, Zhengyu Bai* and Lin Yang**

Supporting Information

Constructing Nanocaged Enzymes for Synergistic Catalysis of CO_2 Reduction

Zhichao Jia, Jianan Dang, Guobin Wen, Yanxing Zhang, Zhongwei Chen,*
Zhengyu Bai, and Lin Yang (s)**

Dr.Z. Jia, Dr.J. Dang, Prof. Z. Bai, Prof. L. Yang
Collaborative Innovation Center of Henan Province for Green Manufacturing of Fine Chemicals, Key Laboratory of Green Chemical Media and Reactions, Ministry of Education， School of Chemistry and Chemical Engineering
Henan Normal University
Xinxiang, Henan 453007, P. R. China.
E-mail: baizhengyu@htu.edu.cn; yanglin1819@163.com

Dr. G. Wen, Prof. Z. Chen
Department of Chemical Engineering, Waterloo Institute for Nanotechnology
University of Waterloo
200 University Avenue West, Waterloo, ON N2L 3G1, Canada.
E-mail: zhwchen@uwaterloo.ca

Y. Zhang
School of of Physics
Henan Normal University
Xinxiang, Henan 453007, P. R. China.

***In situ* ATR-SEIRAS Measurements.**

The ATR-SEIRAS measurements were examined in a two-compartment spectroelectrochemical cell comprising three electrodes, including the working electrode, a platinum wire as the counter electrode, and a standard Ag/AgCl electrode as the reference. All the ATR-SEIRAS spectra were acquired using a Fourier Transform Infrared Spectrophotometer (FT-IR, Shimadzu.). All electrochemical tests were tested in 0.01 M PBS aqueous solution with constant CO_2 flow and controlled by a CHI electrochemical workstation (CHI760E). All spectroscopic measurements were performed at a spectral resolution of 4 cm^{-1}.

Electrochemical measurements.

The electrochemical CO_2 reduction reaction was carried out in a two-chamber electrochemical cell with a Nafion-117 proton exchange membrane between the cells. The carbon cloth was used as the working electrode; the platinum sheet and the Ag/AgCl electrode were used as the counter electrode and the reference electrode, respectively. The electrochemical measurement was performed in the CHI760E electrochemical workstation (Shanghai Chenhua Instrument Co., Ltd., China), 10 mM PBS solution was used as the electrolyte, and the test temperature was 30 °C. The actual enzyme loading of the electrodes is about 2.5 mg/cm^2. High-purity CO_2 was introduced into the electrolyte to maintain a saturated CO_2 environment.

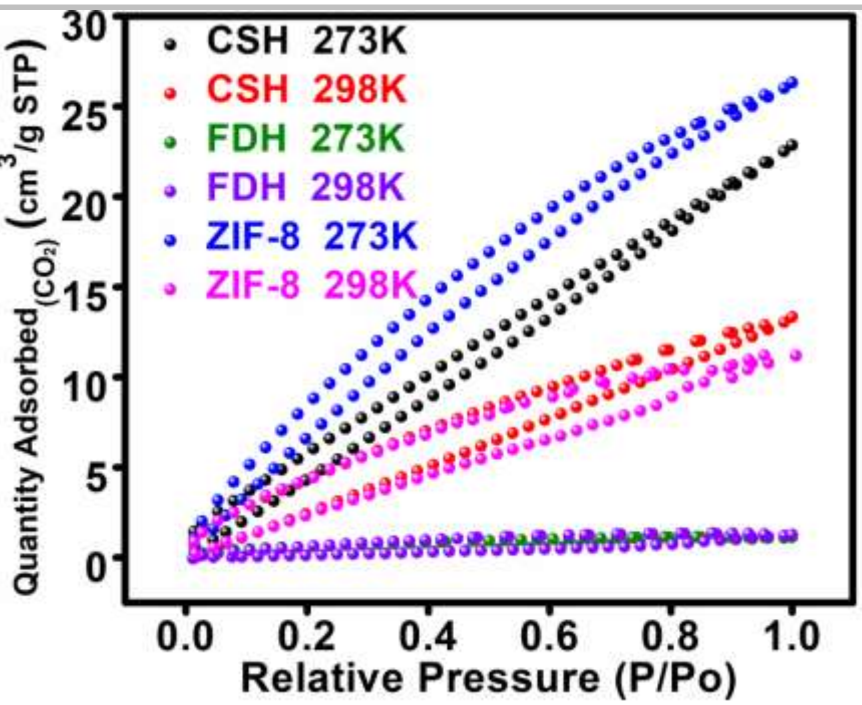

Figure. S1. The CO_2 adsorption and desorption isothermsof FDH, ZIF-8, and the CSH catalyst at 273 K and 298 K.

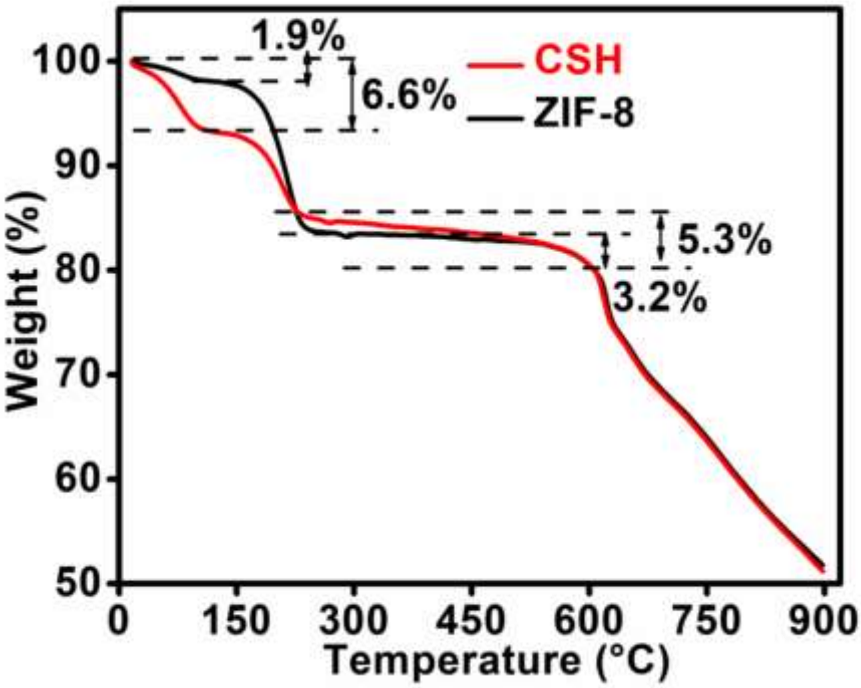

Figure. S2. Thermal gravity analysis of the CSH catalyst and ZIF-8.

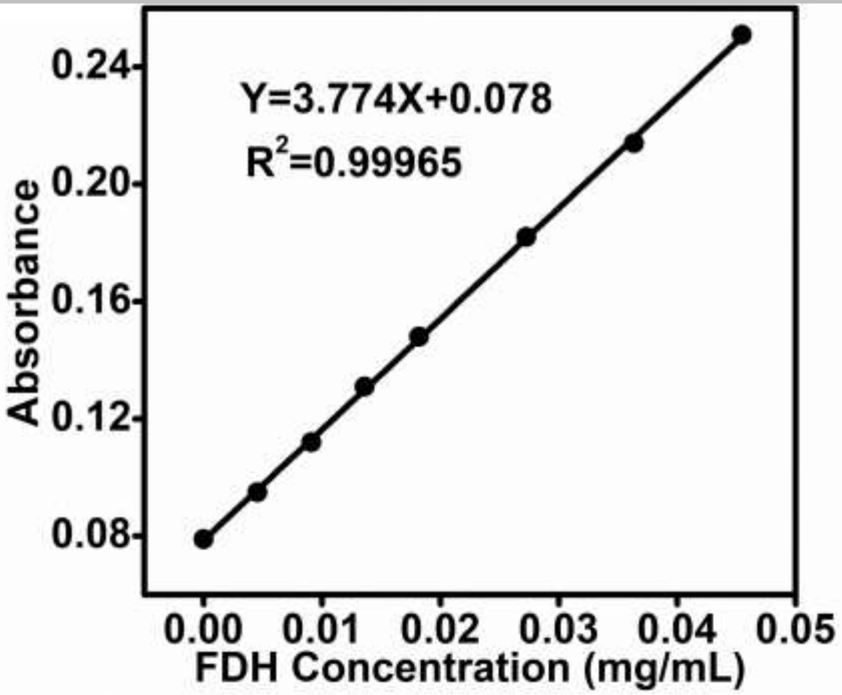

Figure. S3. UV-vis standard curve for FDH concentration.

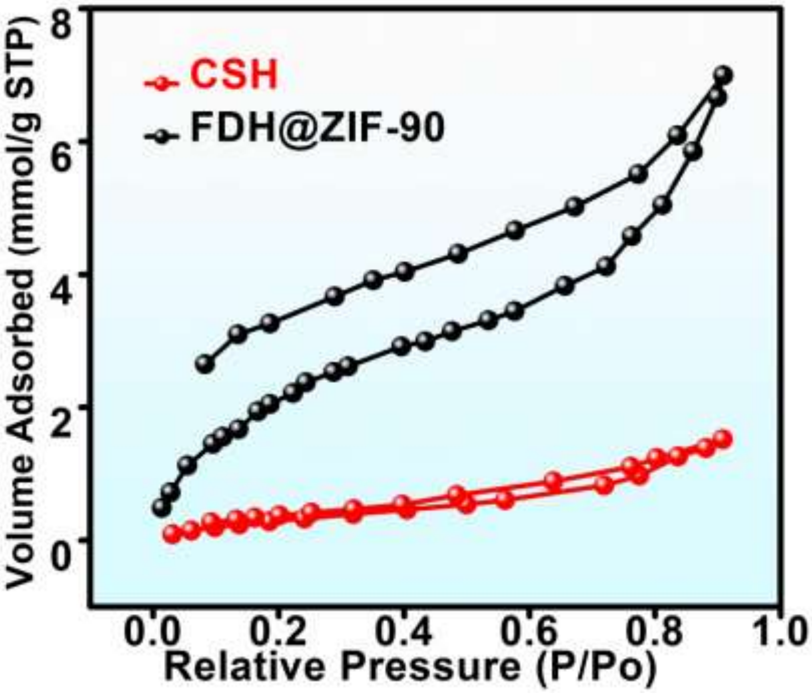

Figure. S4. Water adsorption isotherms of the samples at 25 °C and 1.0 bar.

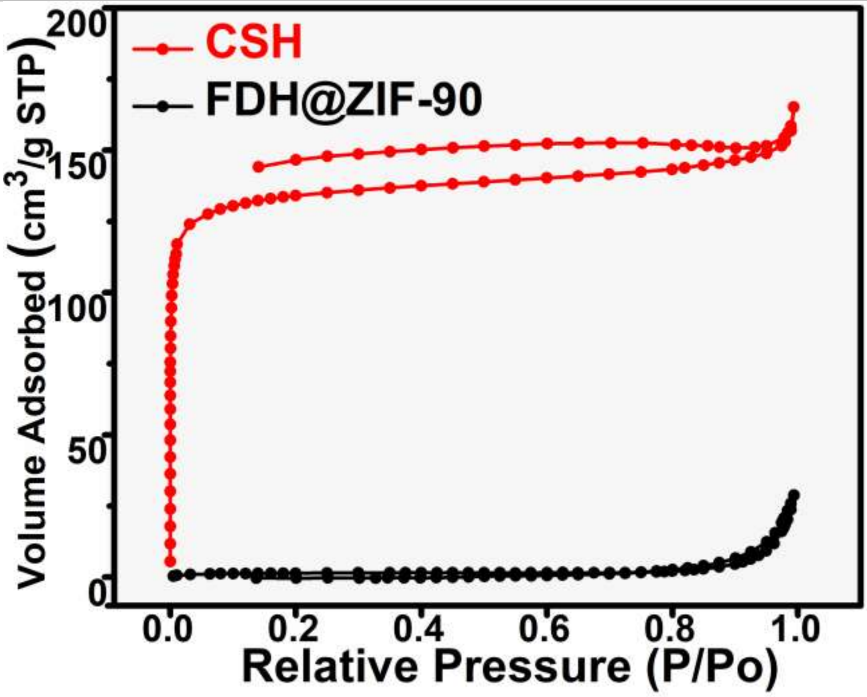

Figure. S5. N_2 adsorption isotherms of the CSH catalyst and FDH@ZIF-90.

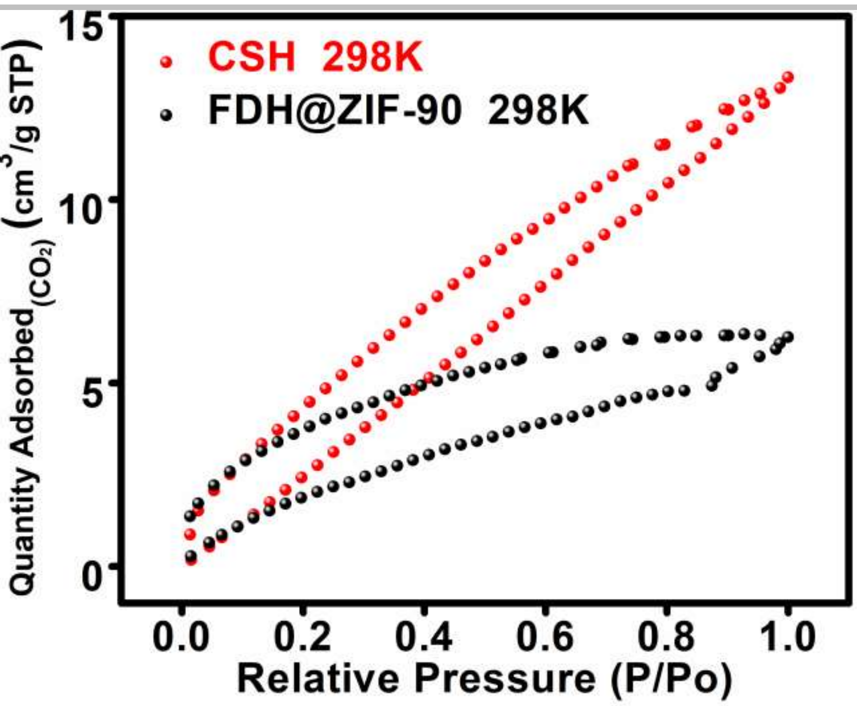

Figure. S6. CO_2 adsorption isotherms of the CSH catalyst and FDH@ZIF-90.

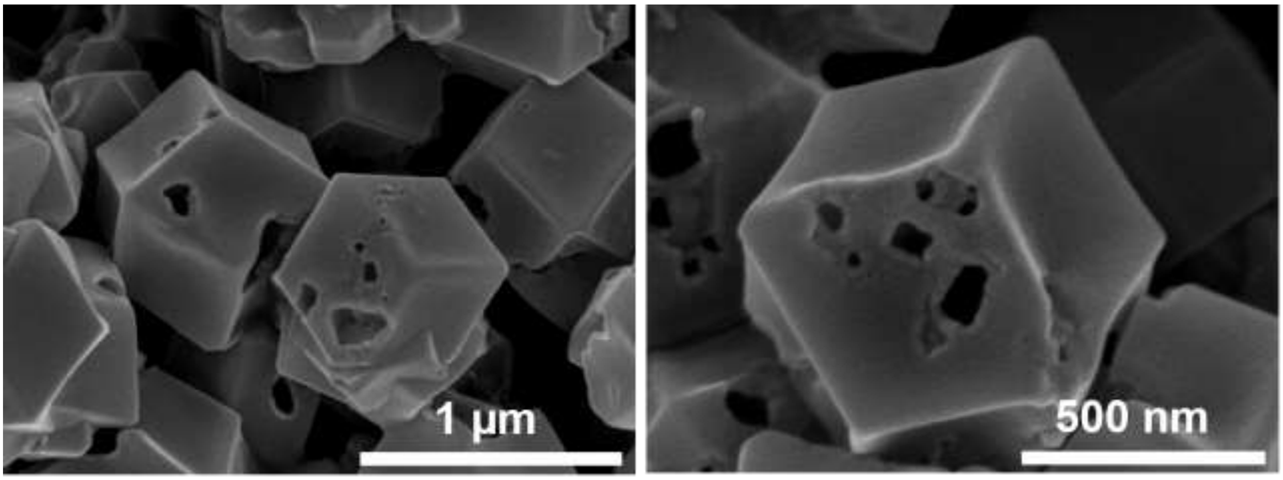

Figure. S7. SEM image of the CSH catalyst.

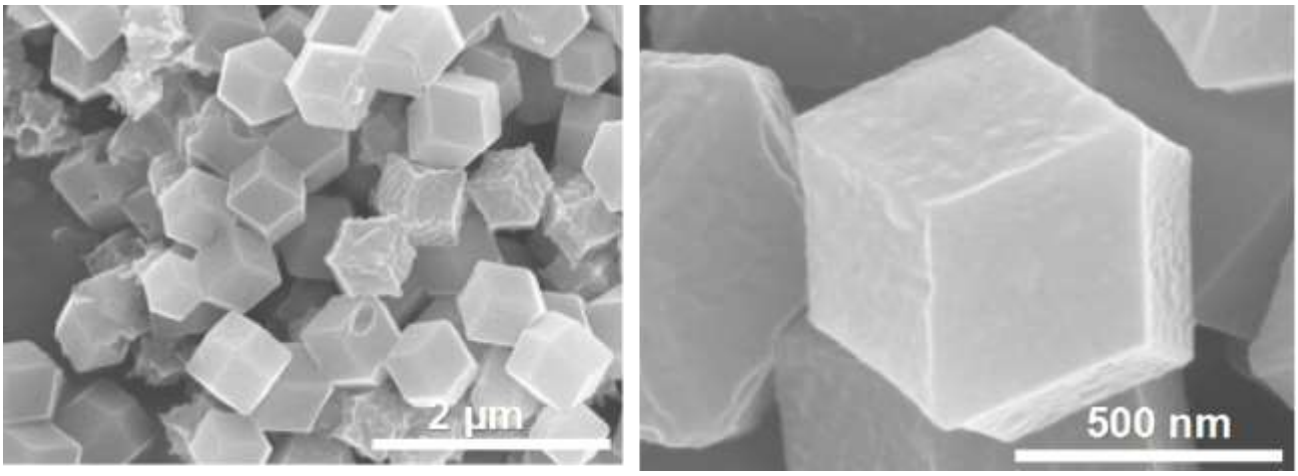

Figure. S8. SEM image of ZIF-8 composites.

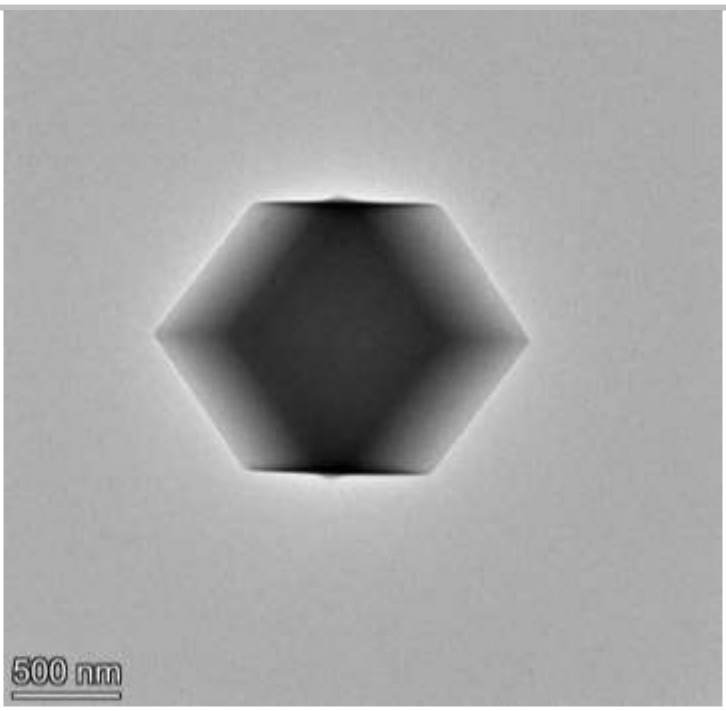

Figure. S9. TEM image of ZIF-8 composites.

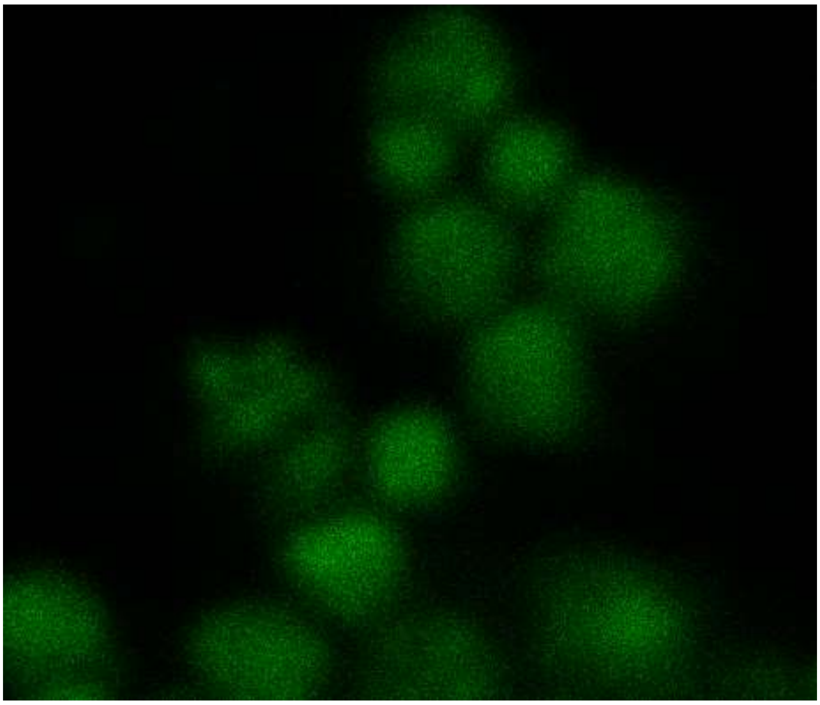

Figure. S10. CLSM diagram of the CSH catalyst.

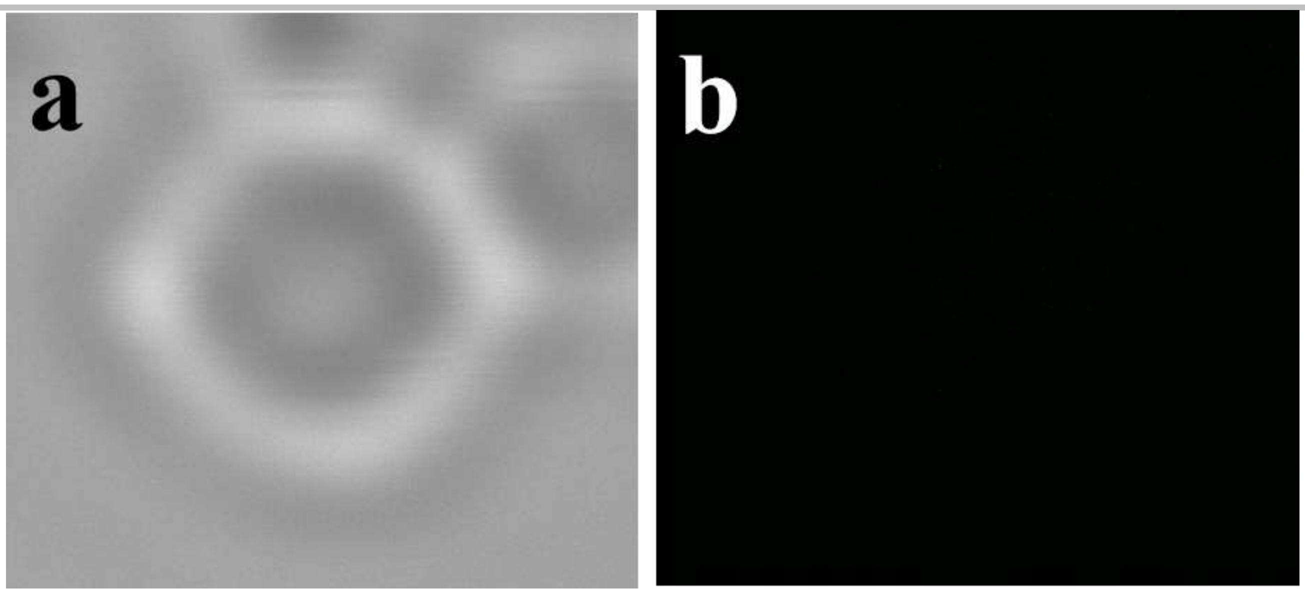

Figure. S11. CLSM diagram of the ZIF-8.

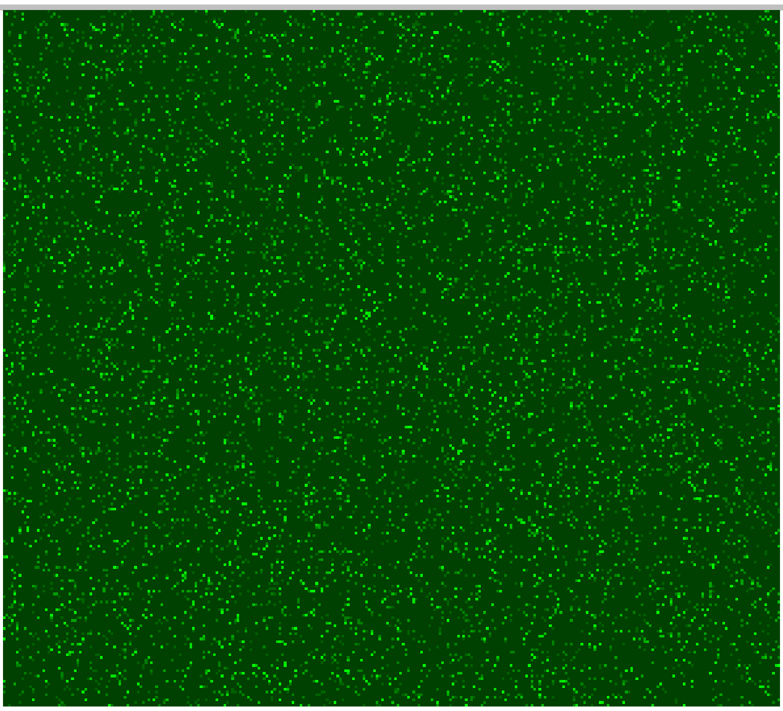

Figure. S12. CLSM diagram of FDH stained with FITC.

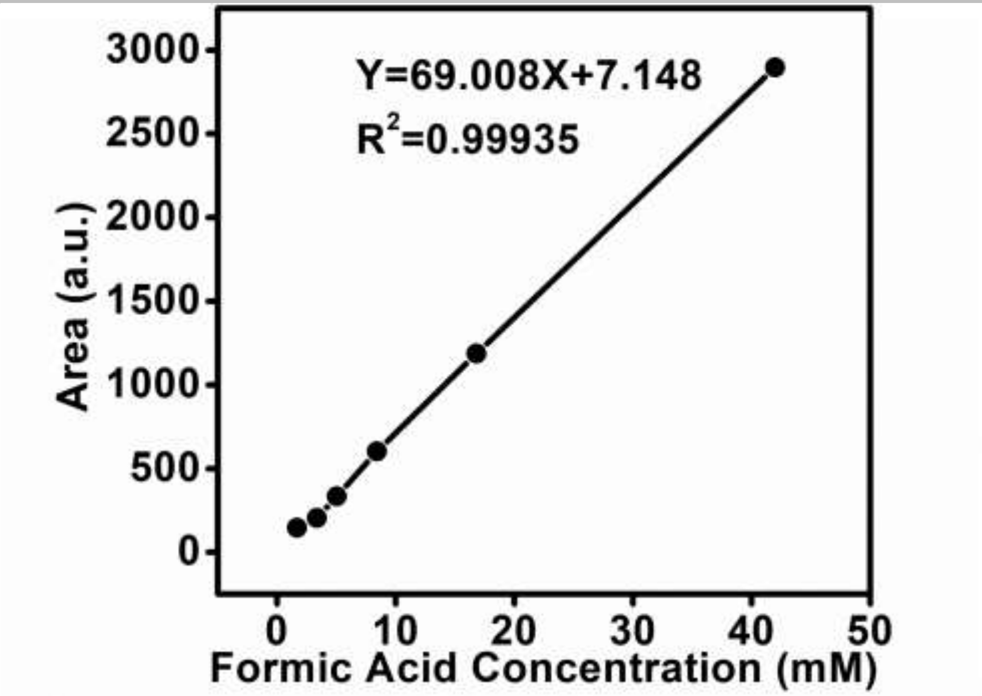

Figure. S13. Standard curve of formic acid concentration in HPLC.

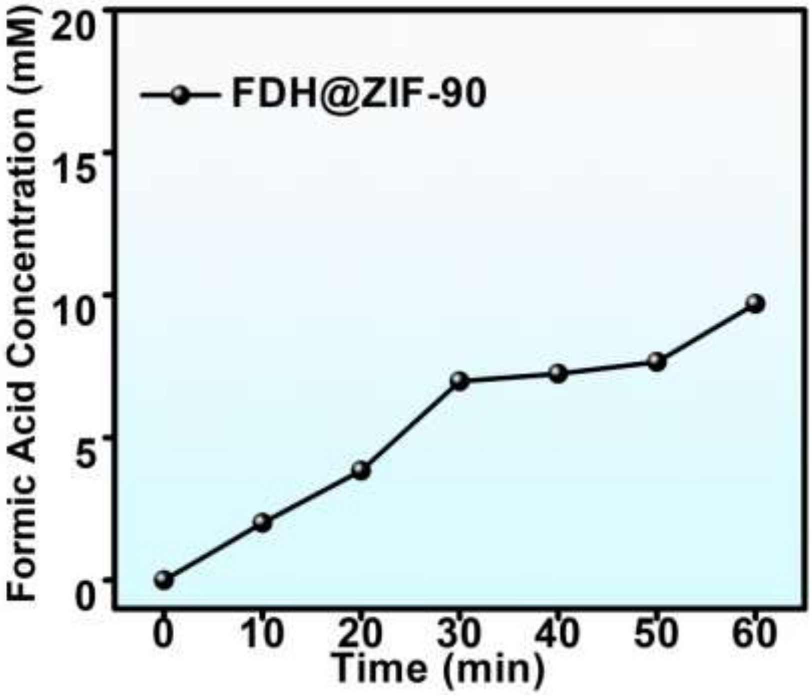

Figure. S14. Concentration of formic acid produced by FDH@ZIF-90 in PBS buffer saturated with CO_2.

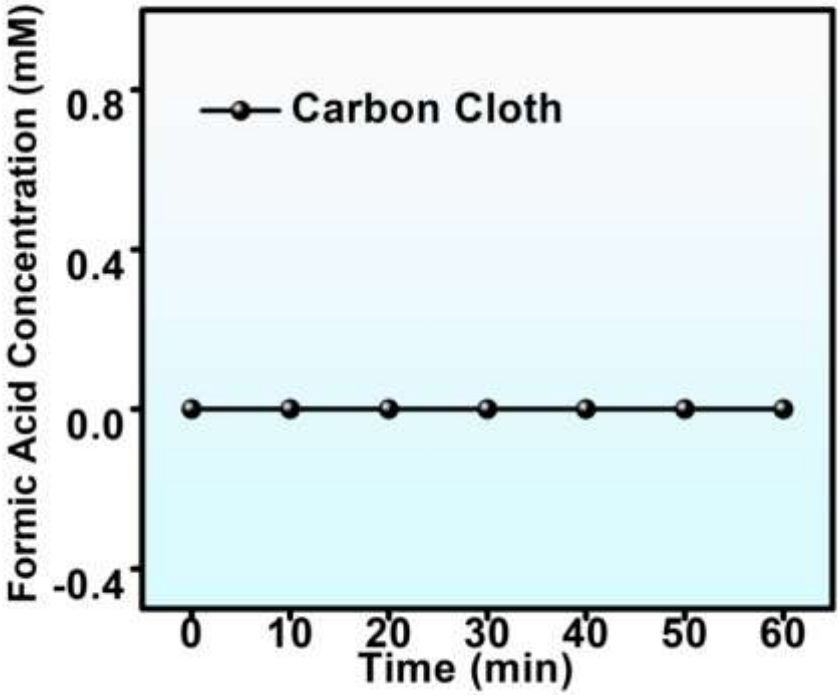

Figure. S15. Concentration of formic acid produced by the carbon cloth in PBS buffer saturated with CO_2.

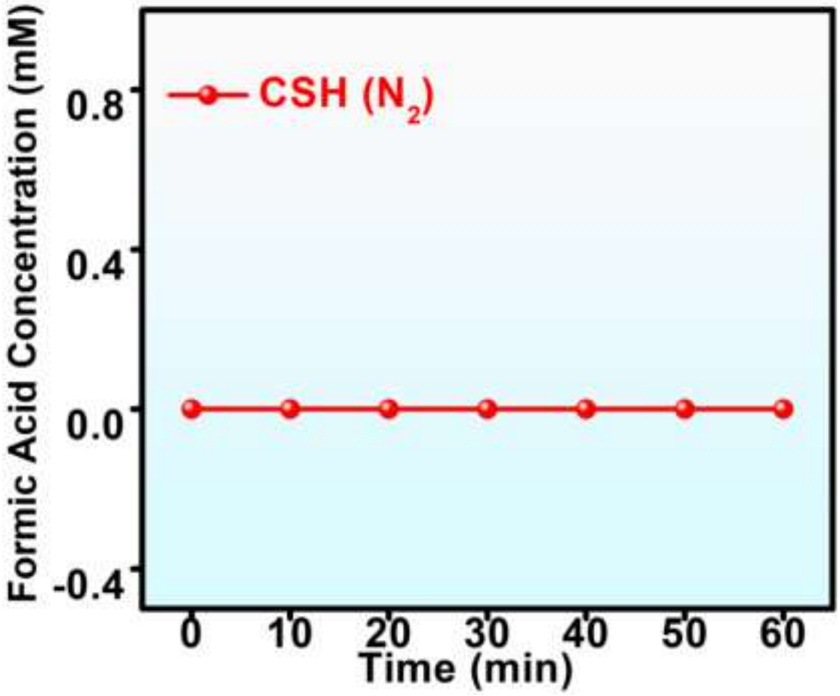

Figure. S16. Concentration of formic acid produced by CSH in PBS buffer saturated with N_2.

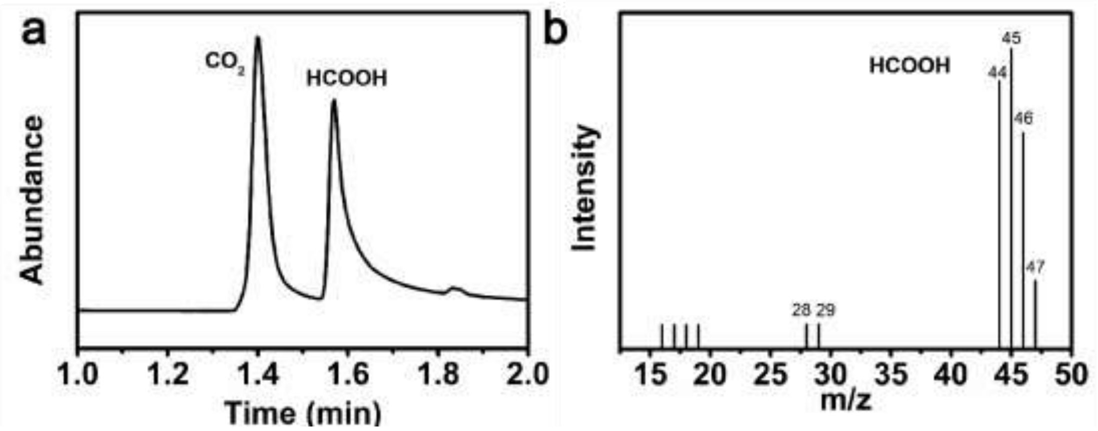

Figure S17. (a) The GC characterization of the electrolyte solutions.
(b) The GC-MS characterization of the electrolyte solutions.

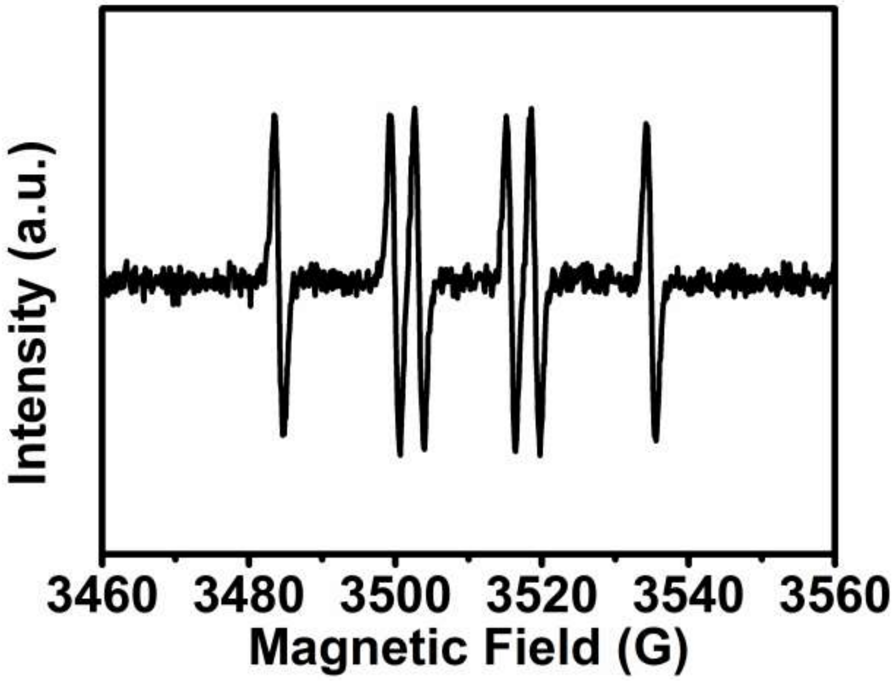

Figure S18. ESR spectra of the electrolyte solutions.

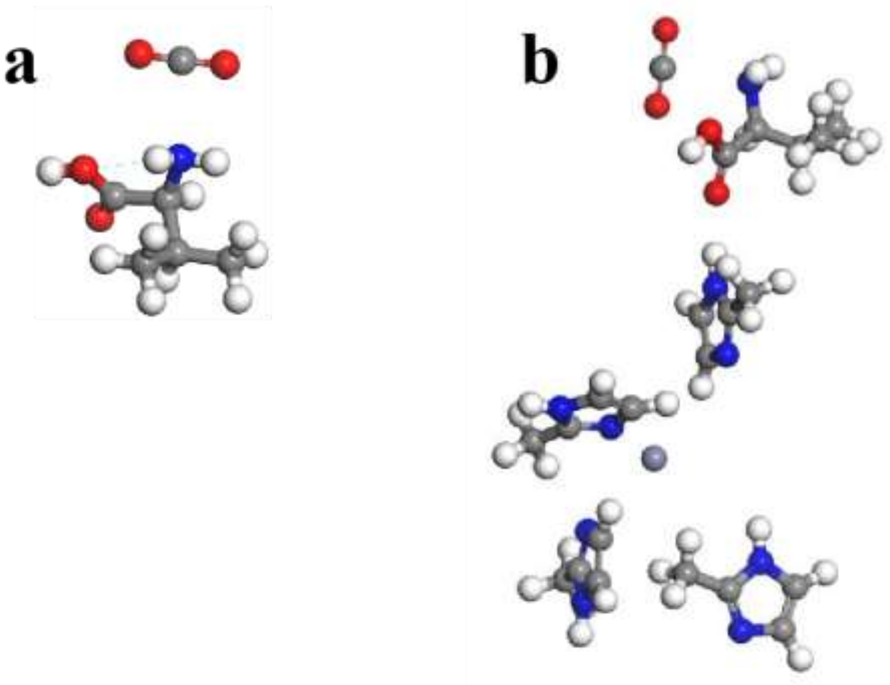

Figure. S19. (a) The adsorption energy of CO_2 on the valine.
(b) The adsorption energy of CO_2 on valine and 2-methylimidazole composite models.

Computational method

The ab initio calculation of the spin polarization with the DFT-D corrections is performed by the Dmol3 code[1]. The PBE functional[2] with Generalized Gradient Approximation[3] (GGA) is used to describe exchange-related interactions. The semi-nuclear pseudopotential[4] (DSPP) is used to replace the nuclear electrons to reduce the computational cost, and the bivalent positive polarization (DNP) basis set is selected. In structural geometry optimization, the energy convergence criterion is 10^{-7} Ha, the force convergence criterion is 0.001 Ha/Å, and the displacement convergence criterion is 0.005 Å. The electronic self-consistency field tolerance is set to 10^{-7} Ha for electronic optimization. To speed up the convergence rate, the stated orbital occupancy rate is 4.7 Å, and the smear value is 0.005 Ha. The Conductor-like Screening Model (COSMO) with a dielectric constant of 78.54 is used to simulate the solvent environment of water. In the electrochemical reduction of CO_2 to formic acid, the reaction steps of CO are as follows.

Formic Acid

$CO_2 + H^+ + e^- + * \rightarrow OCHO*$ (1)

$OCHO* + H^+ + e^- + * \rightarrow HCOOH$ (2)

$CO_2 + H^+ + e^- + * \rightarrow COOH*$ (3)

$COOH* + H^+ + e^- \rightarrow HCOOH*$ (4)

* Stands for the adsorption point on the catalyst. Using the calculated hydrogen electrode, the relationship between the reaction energy of the electrochemical reaction and the potential U and RHE was measured. The change in Gibbs free energy (△ G) of each reaction step is given by the following formula[5].

$\triangle G = \triangle E + \triangle ZPE - T\triangle S + \triangle\int C_v dT + \triangle G_U$ (5)

Here, $\triangle E$ is the reaction heat of a specific reaction step, and $\triangle G_U = -eU$, with U being the electrode potential. $\triangle\int C_v dT$ is the integrated heat capacity; T is temperature, and S is the entropy. Zero-point energies (ZPEs) are calculated with the vibrational frequencies of

adsorbates and molecules as calculated within DFT. The free energy of 1/2 H_2 can replace that of ($H^+ + e^-$) by setting the reference potential to the standard hydrogen electrode.

Author Contributions

L.Y. supervised the project. All authors discussed the results and commented on the manuscript.

References

[1] B. Delley, *J. Chem. Phys.* **2000**, *113*, 7756.

[2] M. Ernzerhof, G. E. Scuseria, *J. Chem. Phys.* **1999**, *110*, 5029.

[3] J. P. Perdew, K. Burke, M. Ernzerhof, *Phys. Rev. Lett.* **1996**, *77*, 3865.

[4] B. Delley, *Phys. Rev. B* **2002**, *66*, 155125.

[5] J. K. Nørskov, J. Rossmeisl, A. Logadottir, L. Lindqvist, J. R. Kitchin, T. Bligaard, H.Jonsson, *J. Phys. Chem. B* **2004**, *108*, 17886.
